# Caregiver and Juvenile Justice Personnel Perspectives on challenges and importance of caregiver engagement and the potential utility of a peer navigator program in the Juvenile Justice System

**DOI:** 10.1186/s40352-023-00231-y

**Published:** 2023-08-05

**Authors:** Allyson L. Dir, Casey Pederson, Shirin Khazvand, Katie Schwartz, Sarah E. Wiehe, Matthew C. Aalsma

**Affiliations:** 1 Department of Psychiatry, Indiana University School of Medicine, Indianapolis, IN USA; 2 Adolescent Behavioral Health Research Program, Indiana University School of Medicine, Indianapolis, IN USA; 3 Department of Psychology, Indiana University Purdue University Indianapolis, Indianapolis, IN USA; 4 Department of Pediatrics, Indiana University School of Medicine, Indianapolis, IN USA; 5 Indiana Clinical and Translational Sciences Institute, Indianapolis, IN USA

**Keywords:** Juvenile justice, Caregivers, Peer support

## Abstract

**Background:**

For youth involved in the juvenile justice (JJ) system, caregiver involvement and engagement in the system is crucial for youth development and outcomes of JJ cases; however, there are challenges to establishing positive/productive partnerships between caregivers and JJ representatives. The current project examines perspectives of caregivers and JJ personnel regarding facilitators and barriers to establishing JJ-caregiver partnerships, as well as their perceptions of the use of a caregiver navigator program to support caregivers of system-involved youth. Results are used to inform development of a caregiver navigator program to support caregivers and help them navigate the JJ system.

**Results:**

Semi-structured interviews were conducted with caregivers of youth involved in JJ (n = 15, 53% White, 93% female), JJ personnel (n = 7, 100% White, 50% female), and JJ family advisory board members (n = 5, 100% Black, 100% female). Caregivers reported varying experiences across intake/arrest, court, and probation processes. Positive experiences were characterized by effective communication and feeling supported by JJ. Negative experiences related to feeling blamed and punished for their child’s system involvement and feeling unsupported. JJ interviews corroborated caregiver sentiments and also illustrated facilitators and barriers to JJ-caregiver partnerships. Both JJ personnel and caregivers endorsed potential benefits of a peer-based caregiver navigator program to provide social, informational, and emotional support.

**Conclusion:**

Continued work is needed to improve JJ-caregiver partnerships and use of a peer-based navigator program has the potential to address barriers to caregiver engagement in the JJ system.

## Introduction

Caregivers play a central role in youth development, and as such, caregiver involvement is a centerpiece in youth service systems, such as education, child welfare, and mental health service systems. The juvenile justice system, however, is one system that has lagged in adopting a family-centered approach (Burke et al., 2014). Historically, the JJ system has operated broadly under the doctrine of *parens patriae*, which allows the state to assume a parental role in order to ensure the safety of the youth and the community (Pennell, Shapiro, & Sprigner, 2011). Nonetheless, in the past few decades the system has come to recognize the important role for caregivers in JJ cases (Walker et al., 2015a; Barnert et al., 2020; Burke et al., 2014). In fact, caregiver engagement and family functioning impacts decision-making by law enforcement and court officials across multiple points of system involvement from decisions at initial arrest (i.e., to detain or release home), to adjudication, and during probation or community supervision (Paik, 2017). It is no surprise that juvenile justice leaders identify family engagement as one of the most important – but challenging – system issues (Development Services Group, 2018). The current study explores caregiver and JJ system personnel perspectives of JJ-family collaboration across multiple points of the JJ system in order to inform the development of a peer-based program to improve caregiver engagement across the JJ system. The manuscript also underscores existing literature highlighting caregiver experiences in JJ.

The Sequential Intercept Model (SIM) outlines the different stages of system involvement from initial contact with law enforcement to initial hearings to community supervision or probation (Munetz & Griffin, 2006). The SIM has been used as a tool to facilitate community-based solutions to support individuals involved in both adult and juvenile justice system (Cintrón Hernández, 2015; Heilburn et al., 2017); it can also be used as an organizational framework to more closely examine different stages of system involvement. The current study focuses on the intercepts of initial arrest / intake, court, and probation because these intercepts require family participation in youth system involvement, unlike detention or placement where youth are removed from the family. Because probation is the most commonly assigned disposition within the system, it is important to capture perspectives on a common opportunity for partnerships between JJ staff and families (Fountain & Mahmoudi, 2021).

## JJ System Perspectives on Caregiver Engagement

Much of the literature on JJ system perspectives of caregiver engagement has focused on JJ system expectations of caregivers and how family characteristics play a role in court and JJ system decision-making.

### Court-level decision-making

Caregiver engagement at the level of court proceedings is important. There has been a long history of courts holding caregivers responsible for youth behavior, and many state laws continue to hold parents responsible and accountable for youth behavior (Harvell et al., 2004). Although caregivers are perceived as passive participants with minimal interaction (Peterson-Badali & Broeking, 2010; Smith et al., 2009), family characteristics and caregiver engagement influence court decision-making. For example, when a family member is present at court, the court is more likely to release youth to the home (Peterson-Badali & Broeking, 2009, 2010). Family characteristics also influence court decisions; for instance, there is evidence that harsher decisions are given at initial arrest and case disposition for youth in single-parent households (Leiber & Mack, 2003; Love & Morris, 2019).

### Community supervision and probation decision-making

There is more available research on system-level perspectives of caregiver engagement at the level of community supervision (i.e., probation); much of this research highlights the challenges of and strategies for establishing collaborative partnerships with caregivers. Commonly reported challenges to engaging caregivers in probation range from practical barriers to complying with probation requirements (e.g., lack of resources, such as transportation) to caregiver attitudes that hinder developing a partnership for probation (Maschi et al., 2013; Schwalbe & Maschi, 2010a, b). One study of probation officers described challenges such as caregivers not understanding or agreeing with the need for probation requirements, as well as caregivers being unwilling to partner with probation officers in the process (e.g., not responding to communications) (Maschi et al., 2013). In another qualitative study, JJ caseworkers recognized how caregivers’ feelings of powerlessness can create a barrier to their active engagement with probation (Sattler & Thomas, 2016).

Other studies examining JJ personnel perspectives have typically focused on JJ personnel expectations for the role of caregivers in JJ cases. In general, across court personnel, probation officers, and police, findings emphasize the importance of caregivers in being present and involved in court or probation meetings and the impact that this has on JJ decision-making. One study identified characteristics of an “ideal parent,” which included caregiver ability to support their child (i.e., provide emotional support, help youth meet court obligations), assert parental authority (i.e., enforce discipline, utilize appropriate parental monitoring), and partner with probation officers in the probation process (i.e., participate in case planning, enforce youth’s compliance with probation requirements) (Maschi et al., 2013). Such characteristics are consistent with other research that has emphasized JJ personnel’s perspectives that caregivers should actively partner with court personnel and provide parental monitoring and supervision (Peterson-Badali & Broeking, 2010).

### Caregiver perspectives

Research on caregiver experiences with the JJ system is also well documented; however, much of the large scale studies of caregiver experiences are dated (e.g., Justice for Families, 2012; Osher and Shufelt, 2006). Also, much of the research on caregivers has focused on understanding parenting practices – such as parental monitoring and support – as it relates to the behavior of their child (Cook, 2013; Cook & Gordon, 2012; Folk et al., 2020; Cavanagh & Cauffman, 2017; Jones et al., 2007).

### Caregivers and the Court

Regarding court experiences, two large national studies have highlighted caregiver experiences of the court process. Across studies, caregivers report that court is associated with emotional stress and feeling blamed for their child’s involvement (Justice for Families, 2012; Osher and Shufelt, 2006). Studies also highlight that court is a rather confusing process and many caregivers do not know what is going on or what to expect in court, which creates additional stress (Hillian & Reitma-Street, 2003; Justice for Families, 2012; Osher and Shufelt, 2006). Lastly, families highlighted that they did not feel involved in the court decision-making process and were not given the opportunity to speak during court or provide feedback (Justice for Families, 2012; Osher and Shufelt, 2006).

### Caregivers and probation

Despite one goal of probation to be a relationship between families and probation officers (Maschi et al., 2013; Vidal & Woolard, 2016), caregivers face many challenges with probation. In a multi-state study examining caregiver experiences with the JJ system, many caregivers felt that probation was a “fast-track” to deeper involvement within the system (Justice for Families, 2012). Across studies, caregivers describe feeling burdened by probation requirements as well as experiencing financial strain that comes with court and probation fees and requirements (Justice for Families, 2012; Models for Change, 2009). Probation involvement can lead to caregivers feeling punished and looked down upon (Luckenbill & Yeager, 2009a, b; Osher & Shufelt, 2006; Ravoira et al., 2012). With that said, one study found that caregivers generally had positive relationships with probation officers, which they characterized as fair, supportive, and helpful towards their children; moreover, positive views of probation officers were associated with parental compliance with probation (Vidal & Woolard, 2016). This is consistent with overarching messages from caregivers regarding the importance of feeling respected and understood by the JJ system (Models for Change, 2009).

System issues such as disproportionate minority contact and structural discrimination as also impact caregivers’ experiences with the JJ system and willingness to engage with JJ personnel (Amani et al., 2018; Fountain & Mahmoudi, 2021). For one, youth of color are significantly overrepresented in the JJ system and are detained at higher rates than White youth (Rovner, 2016). Structural inequalities in the law and the system remain, such that well-intentioned system efforts to focus on family engagement – such as requiring parenting classes – have unfortunately resulted in more blame and responsibility placed on families of color and appear to perpetuate biased treatment (Amani et al., 2018; Piquero, 2008). Other structural barriers that disproportionately impact racial/ethnic minorities make it challenging for youth and families of color to comply with system requirements, in turn leading to deeper system involvement (Fountain & Mahmoudi, 2021). Such inequities contribute to lack of trust and credibility of the system, which further impedes efforts to establish positive JJ-family partnerships (e.g., Amani et al., 2018; Hillian & Reitma-Street, 2003; Vidal and Woolard, 2016).

### Family Engagement efforts

Given the importance and challenges of caregiver engagement, significant efforts to improve engagement have been made (Burke et al., 2014; Walker et al., 2012; Walker et al., 2015a). For example, in an effort to increase caregiver engagement, supplemental grant funding for courts to administer extrajudicial programs is often contingent upon the provision of evidence-based family-based services (Walker et al., 2015a). Other efforts to improve family engagement have included additional training of court personnel as well as implementation of family-centered treatment models that address not only parenting skills but also ecological and environmental factors impacting youth (Walker et al., 2015a; Robertson et al., 2019).

The current study explores the potential use of one such strategy – peer-led system navigators – as a way to increase caregiver engagement with the JJ system. Peer navigation models involve utilizing individuals with shared culture or previous/lived experience navigating systems to provide emotional, social, and informational support to those currently involved in a system; such models have been used in a number of youth-serving systems including mental health and medical systems (Godoy et al., 2019; Lammers et al., 2019; Nayak et al., 2022). Evaluations of family navigator programs have shown promise in use of navigators to increase caregiver social and concrete support (Gottlieb et al., 2016; January et al., 2015), reduce caregiver frustrations with the mental health system (Markoulakis et al., 2018; Myers et al., 2015), and reduce time to service connection (Broder-Fingert et al., 2020; Feinberg et al., 2021). Given the aforementioned challenges that caregivers involved in JJ face, such a model could address some of these issues.

The use of peer navigation models have been implemented in a limited number of jurisdictions in the JJ system. One such program, called Juvenile Justice 101, provides support to caregivers during the court process; a randomized trial of JJ 101 found that use of navigators increased caregivers’ self-efficacy in navigating JJ court processes (Walker et al., 2015b). The Parent Support Program (PSP) was developed in New York City and offers support (emotional, informational, connection to resources) to caregivers across multiple stages of system involvement starting with court; in an unpublished evaluation of the program, they found that caregivers engaged in PSP reported increased sense of agency and understanding of JJ system procedures, and families involved in PSP had better JJ case outcomes compared to those in the control group (i.e., lower rates of out-of-home placements and probation violations) (Impact Justice, 2019). Family Connect targets youth on probation and helping families connect to needed behavioral health services through utilizing trained linkage facilitators (not peers) who had training and knowledge of the behavioral health system (Elkington et al., 2022). A pilot trial demonstrated evidence for feasibility and acceptability of the program (Elkington et al., 2022). Other similar programs, such as use of family advocates, have been utilized in other states like Pennsylvania (Models for Change, 2009); however, empirical research on effectiveness is lacking.

The current study adds to the existing literature on caregiver experiences of JJ involvement (see Justice for Families, 2012) by examining both caregiver *and* JJ personnel perspectives on the facilitators and barriers to establishing collaborative JJ-caregiver relationships at multiple points across the JJ system. Caregiver experiences are documented in the literature (Osher & Shufelt, 2006; Justice for Families, 2012); however, most research has been published over 10 years asgo, and no studies have directly compared caregiver and JJ personnel perspectives. Such research is critical in order to inform needed system-level changes to improve family engagement in JJ. Moreover, the primary goal of the study is to utilize findings in order to inform the development of a caregiver navigator program with the goal of increasing caregiver support and engagement with the JJ system. Interviews provide information on experiences of JJ personnel and caregivers as well as their perspectives on the potential use of a peer navigator program.

## Method

### Sample

Qualitative interviews were conducted with JJ personnel (n = 7), members of a family advisory board (n = 5), and caregivers of youth (n = 15) currently involved in the JJ system. JJ personnel from one Midwestern urban county were recruited to participate using convenience and snowball sampling (n = 1 judge; n = 1 probation officer supervisor; n = 1 intake supervisor; n = 1 probation officer; n = 2 intake probation officers). The authors partnered with an existing family advisory board called FCC that was formed to advise the local juvenile court in their efforts to better understand and improve family experiences with the JJ system. Advisory board members included caregivers of youth previously involved in the JJ system (no caregivers had youth with JJ involvement in the past 5 years) and service providers who work closely with families impacted by the JJ system; all active members agreed to participate in the study. Caregivers of youth *currently* involved in the JJ system were recruited from the urban county (n = 2) as well as two other rural counties (n = 13) in Indiana given the research team’s JJ system connections in these counties. The two rural counties were included in participant recruitment efforts due to low recruitment in the urban county; the COVID-19 pandemic limited access to and availability of participants when the interviews were being conducted. All procedures were approved by the IRB.

### Qualitative interviews

Semi-structured interview guides focused on understanding (1) caregivers’ experiences in the juvenile justice system; (2) relationships between JJ personnel and caregivers; and (3) perspectives on a peer-based program for caregivers to help them navigate the JJ system. Separate interview guides were developed for JJ personnel, caregivers, and fFCC members with overlapping, related questions. Caregiver semi-structured interview guides highlighted facilitators and barriers to navigating the system from initial arrest to adjudication to probation, nature of relationships with JJ personnel, suggestions for improving experiences, and suggestions and opinions regarding a potential peer-based navigator program. System personnel interview guides highlighted perspectives on the role of caregivers in JJ cases, facilitators and barriers to building relationships with caregivers and families, and suggestions and opinions for a navigator program. Interview guides with FCC members addressed perspectives on family engagement in the JJ system as well as perspectives regarding a navigator program. Qualitative interviews with participants were audio recorded and transcribed verbatim for coding.

### Analysis

Transcribed interviews were uploaded to MAXQDA for coding and analysis. We conducted thematic analysis utilizing a phenomenological framework, such that we focused on capturing caregivers’ and JJ personnel’s lived experiences through the interviews (Sundler et al., 2019; Braun & Clarke, 2006). Qualitative codes were developed using a combination of (1) a priori categories based on the research questions and previous research on JJ-caregiver relationships and (2) themes that were identified through inductive review of the interview transcripts. Initial coding by the first author was conducted to develop a set of initial codes. Next, first and second authors conducted additional initial coding with a subset of transcripts to develop a final set of codes. Finally, focused coding with all transcripts was conducted with the first and second author; coders met frequently throughout to ensure reliability. Following coding, demographics of participants were examined for potential patterns in responses, and coding was also examined for patterns in responses from JJ and parents.

## Results

Of the n = 15 caregivers interviewed, 53% (n = 8) identified as White, 47% as Black (n = 7), 6% as Hispanic (n = 1), and 93% (n = 14) as female (caregivers’ youth ages ranged 14–17, *M* = 16 years). All caregivers had already experienced adjudication through the JJ system and the majority of caregivers’ youth were currently on probation. Of JJ personnel (n = 7), 100% identified as White and 50% identified as female. JJ personnel represented intake (n = 1 intake supervisor; n = 2 intake officers), court (n = 1 judge), probation (n = 1 probation officer, n = 1 probation supervisor), and diversion (n = 1 diversion program officer/coordinator). All FCC members (n = 5) identified as Black and female (n = 3 members had children previously involved in the JJ system). There were no differences in patterns of caregiver demographics or responses (i.e., qualitative themes) across urban and rural counties. Below we describe emergent themes related to caregiver and JJ experiences as well as perspectives on the development of a peer-based navigator program for caregivers. Additional quotes highlighting themes are presented in Table 1.

**Table 1:** Highlighted Interview Quotes

Theme	Caregiver / Family Advisory Board Members	JJ Personnel
Intake / Initial Arrest	“It was really scary…like I didn’t know what was going on. I didn’t know. Like I never had this happen before so I’m kind of flabbergasted like I wanted more, I wanted more information.” (caregiver)	“It’s a whole different beast and they’re [caregiver] frustrated and they’re angry. And if the call is coming in at three o’clock in the morning that my child is arrested and is at intake, yeah that’s going to be a little different response.” (probation supervisor)
	“It’s been like a rollercoaster kind of a little bit. It’s been, like, kind of scary, kind of upsetting a little bit, and everything else.” (caregiver)	“It can be at times tense because I’m usually the first person that calls the parents after their child’s been arrested. So, it can be a bit standoffish at times.” (intake officer)
Court	“I feel that she was out for blood and she was trying to make an example out of my son. Don’t get me wrong. He did do wrong and everything. It was his very first time he ever got in trouble for anything and I just feel that she just really socked it to him…” (caregiver)	“It’s really hard for some of these parents. I know the application process to fill out the correct forms and stuff can be a challenge from some parents. They don’t have a lot of experience that way.” (intake officer)
	“You really don’t, you feel absolutely no value from these people that are here to ‘help’. I felt more of a problem and a nuisance, like I was, you know, taking up their time, even though that’s probably what they’re there for, is to help. It didn’t feel like they really wanted to help.”	“I think a lot of parents feel like they’re just getting punished for what their kids [did] and nobody’s really listening to their side of the story.” (intake officer)
Probation	“I think it’s been good. He makes sure that we understand everything and he answers any questions that we have and he’s not pulled no punches or anything. He’s been straightforward about everything.” (Caregiver)	“There’s certain things obviously, kids being juveniles, kids can’t take themselves to the doctor’s appointments and get certain things set up. They can’t enroll themselves in school. There’re expectations that as a parent we would expect that the parent does parental things.” (PO supervisor)
	“She had a lot of different things she had to do, and I kind of felt like it was tough for me too, because there’s some times where I felt a little punished in a way because I had to figure out for her to get all these places, and I do work, and I do have other things to do in my life… For me to try to figure out how to accommodate her and get her here and there was a little difficult.” (caregiver)	“I think there are frustrations having contact with someone with our title, probation officer, as well. I think that they think that’s intrusive and they don’t understand why we’re making contact with them. And then like you said, engagement. Some just don’t want to engage and don’t feel the need to monitor and update us, so that can be a struggle too.” (PO)
	“You feel so defeated. And, you know, when you’re going to juvenile probation, and you know, I’m thinking, ‘Oh, my gosh, finally, you know, maybe these adults will see and they’ll want to help.’” (caregiver)	“Some parents are very involved and compliant and do everything that we ask them to do or the court asked them to do. They attend appointments. They follow up after meetings, etc…A lot of times parents will, they don’t even maintain any communication …It’s all individual depending on the case, depending on the parent.” (probation supervisor)
	“Like I was very resentful about going to see the probation officer, like you taking time out of my days, because I have to bring him to you, I make, have to make family time. So, I mean, they tried to be flexible, like early morning but you know, when you have a job that looks at you like you’re gone again?” (community advisory board)	“I think we have some tough parents… I hear more from the [probation officers] probably frustration in dealing with parents than they do with the kids…they just struggle with how do we tell adults to be the adults in their relationships and to be the parents and the relationships. When they’re trying to work with the kids and they can get the kids on board… we see the light in the kids’ eyes where they’re on board and they get it but the parents not.” (probation supervisor)
	“We had a good relationship. She kept me informed on everything. She would send me text messages when it was time for court…she kept on top of it.” (caregiver)	“I think another barrier is just a parent’s unwillingness sometimes to want to cooperate with what needs to be done and just kind of piggybacks on what I just said about a lot of times, that’s one of the roadblocks that we face, is the parents being the biggest challenge, more so than the child.”
	“Annoying, but she did have like this probation officer that really took an interest in her and, you know, her well-being and trying to help get her on the right path. So that wasn’t a bad experience at all. Like he’s a really great guy. And he suggested some things and gave us some resources. So it’s not always bad.” (caregiver)	
Caregiver Navigator Program	“There’s a lot of navigating through the system and understanding. A lot of our parents that we work with in the community… some of them stop at third grade and they’ve not graduated or they can’t read. And so they have this thing of ‘I’m embarrassed. I’m not going to go in there.’ And so they react and articulate emotions because they’re trying to camouflage the fact that they can’t even read the document. And so let’s get in front of that but you can’t get in front of that until you know them and building that relationship before.” (family advisory board)	“Just the explanation from experience from someone else, I think helps them, because I feel like they are confused and feel like things are just targeted at them. Like ‘you’re calling me for no reason. I don’t understand why you’re making contact with me.’ Things like that, and the navigator could say, ‘No, that’s completely normal. You’re not doing anything wrong. They have to check in.’ So help them understand just the things like that at the beginning.” (probation officer)
	“To have the experience through the justice system to know firsthand, ‘hey, as a parent, this is what my questions were. I didn’t know that I could advocate for this, that I could ask this, that I needed to be able to express the need here.’ So to be able to have that as part of the job description and then training because even though they’ve been through that experience we still need those individuals to look through a different lens as well.” (family advisory board)	“If we can reduce that anxiety and really educate them, that’s great. Then I would say we want [navigators] deeply involved in their child’s case throughout and understanding what services are being ordered and why they are being ordered and also being an advocate for the parent.” (judge)
	“Sometimes parents need that -- and children. They need that surrounding just to let it out. You know, sometimes you’re locked up in the house or work is so hard doing other things so much, you don’t have time to even express that feeling. You know, sometimes you have to get stuff off your shoulder.” (caregiver)	“The parent may open up to [the navigator] more about what they need help with. So maybe if it’s not connecting them directly with the resources, at least telling us, ‘hey, mom’s struggling with family relationships, maybe they need therapy’, and then we can take it from there.” (probation officer)
	“If they’re anything like me they don’t know what’s going on. And you need that kind of advice and -- from somebody that’s already been through it and is familiar with it. Because I believe that it’s, you know, it’s a learning experience.” (caregiver)	“I think probably maybe answer as many questions they have as the parent navigator, what’s going to happen and what to expect. I mean, there is some distrust with the probation officer and the parent.” (probation officer)
	“If dealing with parent to parent, you’d be able to open up I think even more than having someone in the justice system standing over you.” (caregiver	“I think maybe a communicator…I would say the parent themselves don’t always express themselves how they want to portray themselves to the court. Because, whether it’s emotions, frustration, anger, whatever it is… they’re not able to say, so maybe the assistance in being able to say… just kind of a communicator, a translator kind of in a sense.” (probation supervisor)
	“Because who are you to tell me what do to with my kid and family and that kind of thing.” (caregiver)	“I think they would just view it as another leg of probation.” (probation officer)
	“I think there are some parents that wouldn’t appreciate it and that because they didn’t want people in their business.” (caregiver)	“They could make it a little difficult, by maybe involving too much…” (probation officer)

### Caregiver JJ Experiences

#### Initial arrest / intake

Caregivers reported that the experience of intake and learning of their child’s arrest/referral was a time of high emotion and stress, with caregivers describing feelings ranging from frustration and anger to confusion, shock, and concern. Caregivers explained how the process was overwhelming and many voiced confusion and uncertainty as to how to navigate the process. Caregivers also noted feeling as though the initial intake was somewhat intrusive as they were asked a number of personal questions. As one caregiver explained, “I’m somewhat of a private person and I just feel violated with being asked all these questions because my child got into trouble.” Table 1 presents additional quotes.

### Court

Caregivers overwhelmingly described the court experience as a challenging and confusing process. Caregivers noted not knowing what to expect, sometimes not understanding what was going on during the court process, as well as uncertainty in their role during court (e.g., whether they were allowed to speak). For example, one FCC member explained her court experience: “I didn’t know what was happening there…I couldn’t even tell you like how I even knew about court, to be honest.” Others noted feeling judged, undervalued, and as though they were not being heard or given appropriate resources. As one caregiver explained:

I felt like I was being looked down on as a parent. Because you’re always being judged, you know, even if you’re -- because there are a lot of parents out there that just kind of let their kids run wild. I am not one of those. And I felt like I was being met with passive aggressiveness also, when I was there. I did not feel comfortable, I did not feel welcomed.

Other caregivers voiced similar sentiments of feeling judged, undervalued, and unsupported (see Table 1).

### Probation experiences

Caregivers described a range of experiences with probation, with some caregivers describing positive experiences with probation and others describing negative experiences.

*Positive experiences.*

In general, caregivers reporting positive experiences with probation officers described relationships characterized by open communication, respect, and collaboration between probation officers and caregivers to meet family needs (e.g., flexibility with meeting times, requirements). First, open communication included being informed about court and probation expectations and being able to ask questions. One caregiver explained: “We communicated well…he explained everything to where I completely understood. He gave me his card…and he also checked up like once a week to make sure that everything was going well in the household.” Flexibility was also a common factor and included probation officer flexibility and willingness to make accommodations to best fit the needs of the family. For example, some caregivers noted probation officers working with them to find alternative solutions to court requirements during COVID-19 when many programs were not available and being willing to adjust meeting times to meet the family’s needs. Lastly, caregivers endorsing positive experiences highlighted feeling respected by probation officers and feeling as though probation officers were interested in their child’s success. One parent summarized her experience: “The probation department is not your enemy. They’re actually – they want your child to succeed. They’re not your enemy. They’re not trying to fight against you because I know a lot of people look at them negatively.”

*Negative experiences.*

Those endorsing negative experiences most often reported feeling blamed and punished for their child’s involvement with the system and feeling as though probation officers did not take into account family-related barriers to meeting probation requirements. Caregivers described stress and frustration over their role and responsibility in their child’s probation and feeling that many of the probation requirements fell on them. As one caregiver explained:

Ripping and running from work to try to get her to places on time and she’s not, she’s punished, but then again, it’s me being punished. It’s very hectic and stressful. And I don’t think it really helps [my child] because I’m the only one who is straining and struggling, not her.

Other caregivers also felt as though they were being punished for their child’s behavior. One caregiver explained “Like they put it on me, like I’m the fault of the behaviors that happened at school, you know? And that’s where I got this, that blame game that did not sit right with me.” Others reported experiences where probation officers were inflexible and not considering family’s other responsibilities. As one caregiver described:

[they said] they would meet me halfway and [do] what we discussed. But at the end, we didn’t meet halfway with what they said, what was going on, this and that about what they wanted to do. Nothing about how I felt, how he felt, how nobody felt.

Table 1 provides additional quotes.

### JJ Personnel Perspectives

#### Initial arrest / intake and court

JJ intake personnel acknowledged that learning of a child’s system involvement can be seen as a time of crisis for caregivers, but that cooperation and communication is important and plays a role in intake and future court decision-making (e.g., whether to detain or release home, whether to divert or send to court). JJ intake personnel explained that often times it can be difficult to communicate and get information from caregivers, as JJ personnel are often perceived as the “system” (i.e., child welfare system) and thus caregivers are hesitant to provide information. One intake supervisor explained, “Partnering with that parent to get as much information as possible to make a good decision…It can be invasive and uncomfortable, and there are parents that don’t want to cooperate and don’t want us in their business.” Intake and court staff also noted that both the intake and court process can be overwhelming and confusing as they are given a lot of information at one time. As one intake officer explained “Many times it’s almost like they hit a brick wall. Like they’re taking in too much at one time and especially if it’s a family that’s not been through the process before.” Nonetheless, intake and court staff emphasized the importance of caregiver presence during the process. One probation officer explained how parent involvement is most crucial during the pretrial stage before sentencing when they have to conduct an assessment/report: “That point is kind of the turning point, where the parent understands what their expectations are and that’s like the last pretrial step before probation.” As one judge summarized the importance of the caregiver’s involvement in the court process, “If the parent isn’t involved, or the caregiver, those kids don’t fair very well. It’s a participatory sport and we really need that person engaged.”

### Probation

JJ personnel similarly highlighted the importance of caregiver involvement in the probation process as well as facilitators and barriers to partnerships. JJ personnel explained expectations of a caregiver as acting as a partner with the probation officer to monitor youth, which entails communicating with the probation officer about their child’s behavior as well as helping to facilitate engagement in court requirements (e.g., scheduling therapy appointments, providing transportation). As one probation supervisor explained: “We hear a lot of times ‘I’m not the one on probation, so why are you needing this from me?’; No, you’re technically not on probation, but yes, you pretty much are on probation.”

JJ probation officers described facilitators and barriers to successful JJ-caregiver partnerships. Successful partnerships were described as those in which aforementioned expectations are met. In particular, when there is open communication, caregivers understand their role, and caregivers feel heard. Probation officers noted challenges in partnerships when caregivers perceived them as being “intrusive” or “invasive”, not willing to communicate, and when there was disagreement over the youth’s needs (e.g., caregiver not feeling that therapy is necessary although court-ordered). JJ personnel suggested that challenges to probation were often primarily due to caregiver resistance to the process rather than the actual youth’s resistance to the process. As one intake officer explained in reference to caregivers in the intake and probation process, “A lot of the time that’s where we can get a lot of the pushback, the parent’s willingness to be actively involved in the process, because it’s not going to work if they’re not.” Probation officers alluded to system mistrust and misunderstanding of their role as some of the reasons for resistance to partnerships; sometimes, system mistrust is related to the caregivers’ own experiences with the justice system. As one intake officer explained “Some of the kids that we work with, their parents have been through the system as well, and so their response to the system is how they interacted with it when they were in it.” One probation supervisor recognized that the system’s communication of expectations is not always effective:

We would like to have a partnership with the parents. I don’t think a lot of times that it’s seen as that. I think from the court side and the probation officer’s side, I don’t think that we’re real good at building that partnership. It’s ‘you have to do X, Y, Z. This is what your child has to do. These are what the requirements are.’ Instead of trying to develop goals together and working more towards a partnership.

Probation officers also recognized that family functioning and practical barriers that families face can be a barrier to engagement. Probation officers admitted that while their expectation for caregivers is to “act as parents”, this is sometimes a challenge, as there is often family dysfunction and stress. One probation supervisor’s comment summarizes this theme:

We just look at it like we’re only asking you to do two things. Two things that a normal functioning family and normal functioning parents should be able to do. But then on the other side of it now, for that family who may or may not be functioning normally, or whatever normal is, that might be overwhelming. We may be not looking at some of the other factors and some of the other barriers about why they aren’t getting something done.

### Caregiver Navigator Program

Caregivers, FCC members, and JJ personnel were also asked about the acceptability of a potential peer-based navigator program to support caregivers currently involved in the JJ system as a way to improve caregiver experiences and engagement with the system. All participants saw potential benefits of using such a program in the JJ system and highlighted that the program could provide important practical and social support to caregivers.

Participants highlighted the benefit of having someone who had been through the system themselves as a resource to better understand system processes and give advice. One caregiver described the potential benefit of offering informational and practical support: “If they’re anything like me they don’t know what’s going on. And you need that kind of advice and – from somebody that’s already been through it and is familiar with it.” Other caregivers also noted the potential for navigators to offer other general parental support and advice. One caregiver explained “It would be nice to talk to other mothers, fathers about what our children are going through and give any advice that they might have on how to – you know, what do you do to prevent this from happening again?” JJ personnel also noted the potential benefit of navigators in being responsible for providing resources: “If it’s someone that they’ve trusted and they’ve gone through the system… they would probably take it [resources, advice] more from that person than me.”

Participants also noted the potential benefit of offering emotional support given that system involvement is a stressful process. One caregiver explained, “It’s easier for [caregivers] to discuss their issues amongst each other, than to discuss with probation officers and the justice system.” One probation officer similarly described the role of a navigator in providing emotional and informational support: “Being that sounding board. Being that support piece as well as kind of an information [source].” Another FCC member summarized as follows: “Be an advocate. Sit at the table with them.”

Despite the largely positive feedback of the navigator program, both caregivers and JJ personnel cautioned that the program may not benefit everyone. Both caregivers and JJ personnel identified that offering such a program could be perceived as just another court requirement and add additional stress rather than providing support. Caregivers and JJ personnel also cautioned that some caregivers may perceive navigators as intrusive and as trying to tell caregivers how to be parents. For example, as one careviger explained: “There’s some parents…they don’t want to listen to nobody. It’s my child, I will raise them whichever way and nobody could tell me.”

## Discussion

We sought to examine perspectives of both JJ personnel and caregivers of youth involved in JJ to understand relationships between JJ personnel and caregivers and challenges to system engagement, as well as perspectives on the potential utility of a peer-based navigator program to support caregivers in the system.

Findings highlight the potential acceptability and utility of a peer navigator program to support caregivers involved in the JJ system. In general, both caregivers and JJ personnel identified that a parent navigator program could provide all aspects of social support: emotional support (care, empathy trust), instrumental support (provision of concrete assistance), informational support, and appraisal support (affirming or validating support) (Hingson et al., 1990). These perspectives on benefits of the program are consistent with findings from actual evaluations of navigator programs which have shown increases in caregiver self-efficacy and agency in navigating systems (evidence of appraisal and informational support; Walker et al., 2015b), improved social and concrete supports (evidence of instrumental support; Gottlieb et al., 2016; January et al., 2015), and reduced system frustration (evidence of emotional support; Myers et al., 2015; Markoulakis et al., 2018) (emotional support). Despite the largely positive perspectives of the program, both caregivers and JJ personnel did caution that the program may be unwelcome for some who may perceive navigators as meddling in their role as a caregiver or may perceive it as a burden and an additional court requirement. Given these findings, a next step includes developing a program in partnership with the FCC and piloting the program.

Findings also corroborate and replicate previous research regarding caregivers’ experiences with the system. Broadly, both caregivers and JJ personnel agreed that the experience of system involvement depends on the point of contact with the system, with unique considerations at intake or initial arrest, court, and probation. Intake and initial arrest were primarily characterized by frustration, stress, and surprise. Court was largely characterized as a negative experience, with caregivers feeling as if they did not have a voice and judged for their child’s behavior. Probation experiences were more variable, with some reporting positive experiences and others reporting negative experiences. Those who had positive experiences felt heard and supported, while those with negative experiences felt burdened by probation requirements and did not feel the process was helping their child. Such findings are consistent with previous caregiver reports of system experiences (Justice for Families, 2012; Osher and Shufelt, 2006). These findings emphasize the need to continue to closely examine experiences of families during different stages of the JJ system.

The SIM is an ideal tool to utilize to better select and tailor interventions designed to improve caregiver and family experiences and facilitate engagement at these different points in the system. For instance, while interventions to improve family engagement at the time of arrest may include emphathizing with caregiver surprise and finding more agreeable ways to ask personal information (e.g., questionnaire as opposed to a verbal interview), interventions at the court level may include preparing caregivers for their role in court or providing specified time during court for parents to share their perspectives (Walker et al., 2015b). Both caregivers and JJ personnel highlighted potential benefits of a navigator program that could assuage these negative experiences at different points in the system through provision of social support. In fact, there is already evidence for the use of navigators in court (Walker et al., 2015b) as well as as a linkage facilitator during probation (Elkington et al., 2022); findings inform that a navigator that follows families across their system involvement starting as early as the point of initial intake following arrest or referral may be most beneficial.

Caregiver confusion and uncertaintly around navigating the system was common across all points of JJ contact, consistent with previous research (Cleary & Warner, 2017; Cleveland & Quas, 2018; Justice for Families, 2012; Osher and Shufelt, 2006). Indeed, the juvenile legal system utilizes unique terminology and processes that seem separate from other service sections (e.g., school, healthcare) even if intrinsically linked (e.g., school resource officers, high behavioral health need amongst JJ involved youth). However, while caregivers may expect and become practiced in navigating the healthcare system by going to a pediatrician’s office for yearly check-ups or the school system through routine attendance and enrollment, caregivers likely do not expect their child to become involved in the JJ system, and, are thus, unlikely to have or seek out a priori knowledge prior to their child’s arrest. Furthermore, JJ system involvement is a relatively rare occurrence compared to involvement in other child-serving systems, and thus, caregivers likely do not have opportunities to learn from other caregivers, family, and friends how the system works. This highlights a critical role for a peer navigator whose first-hand knowledge is invaluable and consistent with caregiver and JJ personnel perspectives on the benefits of a navigator.

One common theme noted at all points in JJ were experiences of feeling judged, blamed, and punished for their child’s system involvement. In fact, even JJ personnel acknowledged caregivers’ sentiments of blame. These findings are consistent with empirical findings on families’ and system personnel reports of experiences within the JJ system (e.g., Amani et al., 2018; Hillian and Reitsma-Street, 2003; Justice for Families, 2012; Ravoira et al., 2012). It may be helpful to consider the ethics framework of procedural justice, which emphasizes that those going through the system feel heard, are treated with respect, and are given a voice within the system (Hough et al., 2010). Beliefs that the legal system has treated them in a procedurally just manner has shown benefits (e.g., reduced recidivism; institutional compliance) for both adults and youth involved in the system (e.g., Aarons et al., 2015; Beijersbergen et al., 2016; Bierie, 2013; Brown et al., 2019; Kupchik and Snyder, 2009), but to our knowledge this framework has yet to be applied to caregivers with youth involved in the JJ system. Findings from the current study reveal the potential utility in applying a procedural justice framework to caregiver involvement in the juvenile legal system, especially given that caregivers’ views of constructs consistent with a procedurally just judicial system seem to be lacking (e.g., lack of voice and respect for their views). It is possible that user of a navigator to provide such appraisal support may increasweFuture research may benefit from using a procedural justice framework to not only understand caregiver experiences but also guide interventions, such as navigators, to improve caregiver experiences with the system.

Findings also point to the importance that the JJ system places on family engagement and the intersection of caregiver expectations of JJ and JJ expectations of caregivers. Reports regarding JJ expectations of caregivers’ involvement suggested that caregivers were viewed as critical to successful legal case resolution, while on the other hand, caregivers perceived their involvement as a burden and a punishment they received alongside their child. This severe disconnect between JJ personnel and caregivers underscores the critical need for better communication, which was even highlighted by JJ personnel. Interestingly, while JJ personnel and caregivers were not on the same page regarding the expectations they had for each other, they were remarkably consistent in identifying similar barriers for building a strong JJ-caregiver partnership. This finding is encouraging, as this would suggest that the problems are known and understood, allowing for ready identification of potential solutions. Such conflict also speaks to the use of a navigator to serve as a mediator between JJ personnel and caregivers to improve communication and understanding between both parties.

It is also important to note that the majority of caregivers and all FCC members identified as Black, while all JJ personnel identified as White. This discrepancy underscores the disproportionate minority contact and structural discrimination that persists across JJ systems. Many of the themes noted by both JJ personnel and caregivers are, in part, a product of structural discrimination. As such, it is likely that solutions such as a navigator program may allay the negative experiences of some families without addressing the root causes of family disengagement. This points to a potentially important role of the navigators to also serve as advocates of system change and to to bring attention to these system-level issues.

The study is not without limitations. The manuscript is lacking perspectives of actual youth involved in JJ; such perspectives are important to understand JJ’s perspectives on the role of caregiver involvement and how this may impact their own experiences and their case. We sampled individuals from two rural counties and an urban county. Although our results revealed similar experiences across jurisdictions, contextual system differences may have influenced responses. Though the small sample size may limit generalizability, we did achieve thematic saturation through qualitative interviews. Additionally, when considering improvements in caregiver engagement, it is important to ensure that solutions are tailored to local, population-specific characteristics. For the current study, perspectives from advisory board members, caregivers involved in the system, and JJ personnel from the urban county – although limited in number – provide important first-hand information for development of the program in this jurisdiction. All JJ personnel were White while the majority of caregivers and all FCC members were Black; while this reality may limit generalizability, our sample was representative of the populations of interest. Future research should explore how race and ethnicity of JJ personnel and caregivers impact experiences and relationships.

## Conclusion

The manuscript adds to the important topic of family engagement in JJ by examining both caregiver and JJ perspectives on the JJ system and exploring perspectives regarding a peer navigator program to provide additional support to families involved in the JJ system. Findings provide support for acceptabuility of a navigator program and the potential for navigators to offer key elements of social support at multiple points across the system in order to assuage many of the challenges that caregivers face. Findings also reiterate the challenges that caregivers face at multiple points in the system as well as structural inequalities that exist that must also be addressed. Such results add to the evidence for the need for more work and efforts to understand ways to improve partnerships between families and JJ better support families involved in JJ.

## Data Availability

The datasets generated and/or analyzed during the current study are not publicly available due to the nature of the data being qualitative interviews but are available from the corresponding author on reasonable request.
